# Tympanic Membrane Perforation among Patients Presenting to Department of Otorhinolaryngology of a Tertiary Care Hospital: A Descriptive Cross-sectional Study

**DOI:** 10.31729/jnma.7269

**Published:** 2022-03-31

**Authors:** Bibek Ghimire, Meenakshi Basnet, Gyan Raj Aryal, Nischal Shrestha

**Affiliations:** 1 Department of Otorhinolaryngology-Head and Neck Surgery, Nobel Medical College Teaching Hospital, Biratnagar, Morang, Nepal; 2 Department of Internal Medicine, Nobel Medical College Teaching Hospital, Biratnagar, Morang, Nepal

**Keywords:** *eardrum perforation*, *hearing loss*, *otitis media*

## Abstract

**Introduction:**

Intact tympanic membrane acts as a partition between the external ear and middle ear. Tympanic membrane perforation is one of the commonest causes of conductive hearing loss. Perforation size is the most important determination of hearing loss. The aim of this study was to find out the prevalence of tympanic membrane perforation among patients presenting to the otorhinolaryngology department of a tertiary care hospital.

**Methods:**

A descriptive cross-sectional study was carried out in the Department of Otorhinolaryngology and Head Neck Surgery of a tertiary care hospital from March 2021 to August 2021. Ethical approval was taken from Institutional Review Committee (Reference number: 57112021). Convenience sampling was done and data was collected from 414 patients presenting to the department. Collected data were entered, analyzed in Statistical Package for the Social Sciences version 21.0 and documented for study. Point estimate at 95% Confidence Interval was calculated along with frequency and percentage for binary data.

**Results:**

Among 414 patients, tympanic membrane perforation was seen in 100 (24.15%) (20.02-28.27 at 95% Confidence Interval). Among 100 patients with a total of 153 perforated eardrums, mild hearing loss was seen in the majority of the cases. Posterior perforation had a mean hearing loss of 40.41±5.96 dB, central had 39.09±3.13 dB, and anterior had 35.15±5.88 dB.

**Conclusions:**

Our study showed the prevalence of tympanic membrane perforation to be high when compared to other similar studies. Hearing loss was observed in all cases; the majority with mild hearing loss. The degree of hearing loss was more in larger and posterior perforation.

## INTRODUCTION

The tympanic membrane (TM) or Eardrum acts as a partition between the external ear and middle ear. It converts and amplifies air vibration. So, tympanic membrane perforation (TMP) can lead to conductive hearing loss.^1^ According to World Health Organization (WHO), pure tone audiogram up to 25 dB is considered normal, whereas, hearing loss of 26-40 dB is considered mild, 41-60 dB is moderate, 61-80 dB is severe, and >80 dB is considered a profound hearing loss.

Tympanic membrane perforation is one of the most common causes of hearing loss. In Nepal, chronic otitis media is the most common cause leading to TMP causing hearing loss followed by trauma and acute otitis media.^2^

Thus, the aim of this study was to find out the prevalence of tympanic membrane perforation among patients presenting to the otorhinolaryngology department of a tertiary care hospital.

## METHODS

A descriptive cross-sectional study was carried out in the Department of Otorhinolaryngology and Head Neck Surgery, Nobel Medical College Teaching Hospital, Biratnagar, Nepal over a period of six months from 2021 March to 2021 August after taking ethical clearance from the Institutional Review Committee (Reference number: 57112021). All the patients within the age group of 11-70 years were included in this study after taking informed consent. Patients with active chronic otitis media squamous type, active chronic otitis media mucosal, acute suppurative otitis media, traumatic perforation, mixed hearing loss, otosclerosis, granulomatous disease were excluded.

The sample size was calculated by using the formula,

n = (Z^2^ × p × q) / e^2^

  = (1.96^2^ × 0.54 × 0.46) / 0.05^2^

  = 381

Where,

n = minimum required sample size
Z = 1.96 at 95% Confidence Interval (CI),
p = prevalence of tympanic membrane perforation as 54.85% based on study a previous^3^
q = 1 - p
e = margin of error, 5%

Hence, the required minimum sample size was 381. However, a sample size of 414 patients was taken for this study and a convenience sampling method was used.

A detailed history was taken and the patient was examined thoroughly. Among 414 patients, diagnosis of chronic otitis media inactive mucosal type was made clinically. Pure tone audiometry, tympanometry, and endoscopy (4x175 mm, karl storz) were done for all the cases. The average air conduction threshold at 500, 1000, and 2000 Hz was calculated for the degree of hearing loss and was graded according to the WHO classification of hearing impairment.

The tympanic membrane was divided into four quadrants by using two imaginary lines, one passing through the handle of the malleus and another line passing through the umbo by making an angle of 90 degrees with the first line. Perforations were classified into three groups based on the involvement of quadrants. Small perforation involved only one quadrant, medium perforation involved two quadrants, and large perforation involved three or four quadrants. Perforation occupying area anterior to handle of malleus of pars-tensa were labeled as anterior perforation and perforation posterior to that as posterior perforation. Perforation occupying area on either side of the handle of malleus on pars-tensa was leveled as central perforation.

Collected data were entered, analyzed in Statistical Package for the Social Sciences version 21.0 and documented for study. Point estimate at 95% Confidence Interval was calculated along with frequency and percentage for binary data.

## RESULTS

**Figure 1:**
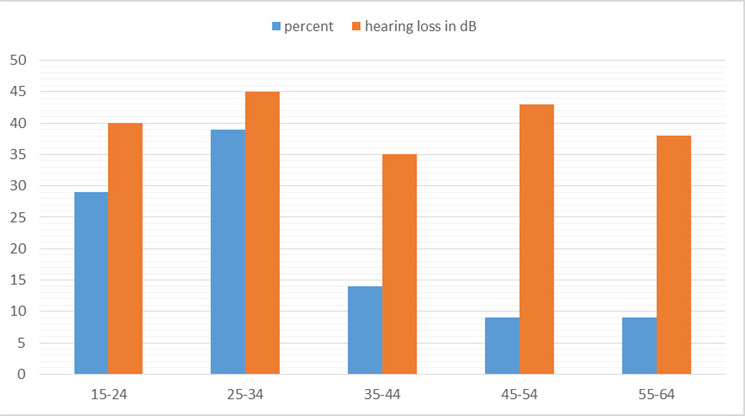
Hearing loss according to different age groups.

Among 414 patients, tympanic membrane perforation was seen in 100 (24.15%) (20.02-28.27 at 95% Confidence Interval). These 100 individuals aged 1170 years (mean age 33.60±14.21 years) with 153 perforated eardrums were then studied. Among these patients, 36 (36%) were males and 64 (64%) were females. Fifty-three (53%) patients had bilateral TMP and 47 (47%) patients had unilateral TMP. The age group 25-34 years had an average hearing loss of 45 dB, which was maximum among the other age group but also accounts for 30% of the total study population (Figure 1).

In all cases of tympanic membrane perforation, hearing loss was documented. In right ear perforation 67 (67%), mild hearing loss was observed in most cases followed by moderate and severe hearing loss. No profound hearing loss was documented. Same findings were seen with left ear perforation (total 86). It was observed that hearing loss in posterior perforation was more than central and anterior perforation (Table 1).

**Table 1:** Degree of hearing loss according to the site (n = 153).

Right ear perforation (n = 67)	Average Hearing Loss (Mean±SD) (dB)
Anterior	35.15±5.88
Central	39.09±3.13
Posterior	40.41±5.96
**Left ear perforation (n= 86)**	**Average Hearing loss (Mean±SD) (dB)**
Anterior	36.30±5.54
Central	38.76±3.06
Posterior	40.32±6.16

It was observed that hearing loss is more in large perforation than in medium and small size perforation (Table 2).

**Table 2:** Degree of hearing loss according to the size of perforation (n= 153).

Right ear perforation (n= 67)	Average Hearing loss (Mean±SD) (dB)
Small	28.83 ± 4.62
Medium	36.20 ± 2.69
Large	41.08 ±3.98
**Left ear perforation (n= 86)**	**Average Hearing loss (Mean±SD) (dB)**
Small	31.50 ± 4.37
Medium	35.73 ±3.18
Large	41.15 ± 3.80

## DISCUSSION

Tympanic membrane perforation (TMP) is one of the commonest causes of hearing loss. The main cause is a secondary infection due to upper respiratory tract infection and poor hygiene in developing countries. TMP can occur from injuries occurring due to ear picking habits, probing, syringing, etc. Tympanic membrane perforation causes conductive hearing loss in most cases but tympanic membrane perforation due to head injuries, blast injuries, etc. may cause inner ear injury leading to sensory neural hearing loss.^2^

This study showed the prevalence of TMP to be 24.15% which is very high compared to the other studies which have shown 2.1%^4^ and 7.8%.^5^ All the cases of TMP in this study presented with hearing loss which is similar to the another study.^6^ In this study, greater hearing loss was seen with the increase in the size of perforation which was comparable to other studies.^7^ In a series of studies done to assess middle ear function with tympanic membrane perforations, perforation size was the most important determination of hearing loss.^8,9,10^ In this study, posterior perforation had a greater degree of hearing loss compared to central and anterior perforation which held true to other study as well.^11^ Same result was seen in a 100 cases study done in 1993.^12^ But some studies stated that there is no significant variation in hearing loss according to location.^13^

Small sample size and sampling bias could be the key limitation of this study. Further, this is based on data collected in a single hospital which limits the universality of the findings. Also, the sampling was done during the COVID pandemic which limited the data collection.

## CONCLUSIONS

The prevalence of TMP was high compared to other similar studies; however, the prevalence of hearing loss in TMP cases and degree of hearing loss according to the site and size was similar to other studies done in similar settings.
